# Dynamic Interface Pressure Monitoring System for the Morphological Pressure Mapping of Intermittent Pneumatic Compression Therapy

**DOI:** 10.3390/s19132881

**Published:** 2019-06-28

**Authors:** Shumi Zhao, Rong Liu, Chengwei Fei, Dong Guan

**Affiliations:** 1 Institute of Textiles and Clothing, Hong Kong Polytechnic University, Kowloon, Hong Kong SAR, China; 2 Department of Aeronautics and Astronautics, Fudan University, Shanghai 200433, China; 3 College of Mechanical Engineering, Yangzhou University, Yangzhou 225127, China

**Keywords:** soft sensor, intermittent pneumatic compression, morphological pressure mapping, lower limb, compression therapy

## Abstract

Intermittent pneumatic compression (IPC) is a proactive compression therapeutic technique in the prophylaxis of deep vein thrombosis, reduction of limb edema, and treatment of chronic venous ulcers. To appropriately detect and analyze biomechanical pressure profiles delivered by IPC in treatment, a dynamic interface pressure monitoring system was developed to visualize and quantify morphological pressure mapping in the spatial and temporal domains in real time. The system comprises matrix soft sensors, a smart IPC device, a monitoring and analysis software, and a display unit. The developed soft sensor fabricated by an advanced screen printing technology was used to detect intermitted pressure by an IPC device. The pneumatic pressure signals inside the bladders of the IPC were also transiently collected by a data acquisition system and then transmitted to the computer through Bluetooth. The experimental results reveal that the developed pressure monitoring system can perform the real-time detection of dynamic pressures by IPC and display the morphological pressure mapping multi-dimensionally. This new system provides a novel modality to assist in the effective evaluation of proactive compression therapy in practice. The study results contribute to understanding the working mechanisms of IPC and improving its functional design based on intuitive biomechanical characteristics of compression delivery profiles.

## 1. Introduction

Compression therapy has become a mainstay for prophylaxis and the treatment of chronic venous insufficiency (CVI) of the lower extremities [1]. An increase in the prevalence of venous disorders such as varicose veins, deep vein thrombosis (DVT), leg ulcers, lymphedema, and blood clots has induced the growth of the global compression therapy demands. The compression therapy market size was valued at USD 2.9 billion in 2016 and is expected to witness a lucrative compound annual growth rate of 5.2% from 2018 to 2025 [2]. The conventional compression stockings and bandages, as the typical static compression modalities, have demonstrated their efficacy in reducing venous hypertension and improving venous return [3,4]. However, high noncompliance, short-term usability, and the lack of flexibility in pressure dosage delivery have limited the applications of compression stockings and bandages in practice. To overcome these shortcomings and enhance personalized treatment efficiency, the dynamic compression technology has been gradually used increasingly since 2015 owing to its benefits including proactive pressure control, high venous return and sustained effect.

**Figure 1:**
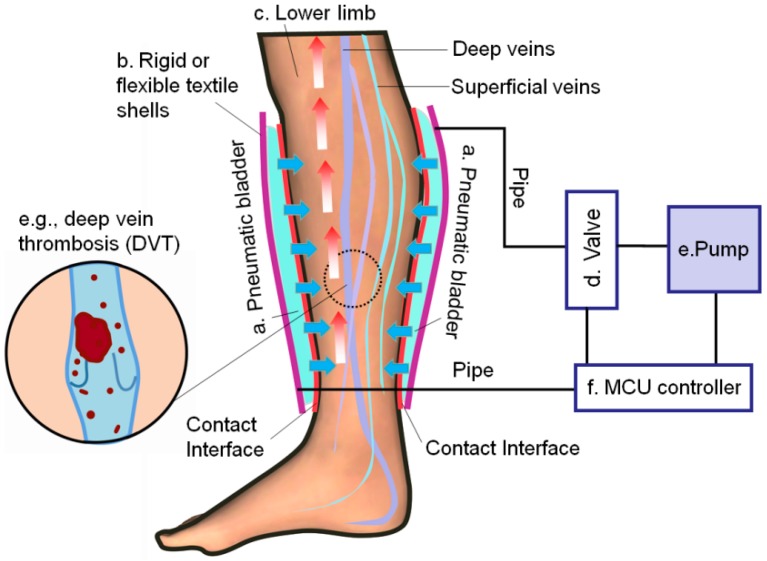
A schematic diagram of the typical intermittent pneumatic compression (IPC) device in compression therapy of lower extremity.

Intermittent pneumatic compression (IPC) is a type of increasingly used dynamic compression therapeutic techniques that are applied to improve venous circulation in the limbs of patients with CVI, edema, or the risk of DVT or pulmonary embolism [5,6]. An IPC electromechanical device was introduced in the early 1970s [7], and further improved in clinical trials [6,8]. The clinical efficacies of the device have been reported by a number of studies in the fields of vascular surgery, dermatologic surgery, and sports engineering [9,10,11,12]. IPC devices are commonly equipped with a single or multiple pneumatic chambers (bladders) that are used to generate different compression cycles by controlling the air in the inflation‒deflation modes [13,14,15]. The typical structure of IPC applied in the lower limb treatment is illustrated in Figure 1. The structure involves (a) inflatable bladders laminated with (b) rigid or flexible textile shells acting on (c) the target lower limb. The compression cycles and pressure delivery modes (e.g., peristaltic centripetal pressure or sequential gradient pressure) generated by IPC were controlled by using (d) a connected air valve, (e) pneumatic pump, and (f) microcontroller unit (MCU). The MCU sends on‒off command signals to the valves and the pneumatic pump based on the control logic. The inflatable built-in pneumatic bladders exert intermittent mechanical forces on the leg skin and tissues for improving hemodynamics. However, till to date, how to effectively monitor, record, and display real-time dynamic pressures by using an IPC device remain less elaborated. This fact has restrained the understanding of the interaction mechanisms of the IPC-lower limb system and the creation of new dynamic pressure alternatives.

Ferraresi et al., established a mathematical model to numerically analyze the working correlations among the involved multicomponent factors (such as air pressure, bladder pressure, tissue pressure) [16,17]. The reliability of the developed model was not validated based on the experimental data. Rithalia et al., systematically analyzed the eight types of intermittent pneumatic compression systems with pressures ranging from 6 to 124 mmHg and the rate of inflation was from less than 1 to 9 mmHg per second [11]. This implied that the variations in the pressure dosages by IPC might be caused by the inflation pressure settings, the volume of the pressure garment, the resistance provided to the flow by the connecting tubes, and the air flow capacity of the pump. However, the existing IPC system cannot present dynamic pressure mapping in a three-dimensional (3D) view when it is placed on lower limbs with irregular shapes. Moreover, the quantitative interactions among pressure dosages varying with inflation and deflation cycles, air mass flow rate, and morphological pressure profiles are not yet fully investigated. Moreover, the optimal working modes for compression treatment for a specific patient with diverse symptoms [18,19] are still unidentified. In general, the intensities of the applied pressures and the rate of inflation are the most important characteristics that should be considered while designing or selecting an appropriate IPC product. Timely detection and quantification of the skin pressure and pneumatic pressure provided by the pneumatic pumping controller system contribute to enhancing our understanding of the design characteristics and drawbacks of the engineered IPC device.

In addition, variations in the biomechanical properties and deformations on the human skin induced by different external forces have attracted increasing attention in fields of electronics, biology, and biomedicine [20]. Dagdeviren et al. developed conformal and piezoelectric devices to measure soft tissue viscoelasticity in the near-surface regions of the epidermis [21]. The study demonstrated the applicability of the system in detecting the contact between a complex topographic surface and the texture of the skin or other organs under both quasi-static and dynamic conditions. Lee et al. developed a pressure sensor fabricated using composite nanofibers of carbon nanotubes and grapheme to reduce the bending sensitivity for a curved surface test [22]. Mishra et al. proposed a bioelectronic system to record high-fidelity electrooculogram by using a mechanically comfortable sensor [23]. Wang et al. developed a novel stretchable and self-healable electronic composite material sensor to evaluate the ultra-sensitive strain and pressure sensing performance of electronic skin [24]. However, the aforementioned studies are related to pressure or biological features that are detected at specific skin regions rather than a real-time dynamic interface pressure monitoring in a continuum skin area. Additionally, whether the interface biomechanical force monitor and display can be used to conversely control pneumatic pressure and skin contact pressure of the IPC-lower limb system is still not elaborated.

Due to the increasing awareness of personalized medicine, the latest wearable device for monitoring and therapy require more intuitive and sophisticated interfaces to be in contact with the body for communication, sensing, and biofeedback [25,26]. Different types of soft pressure sensors have been developed by using nanomaterials coupled with flexible and stretchable polymers [27,28]. Flexible piezoresistive sensors comprising conformable substrates and compliant conductive materials are capable of detecting the applied pressure or mechanical force through changing current or resistance. Some advanced materials are used to fabricate highly flexible and stretchable strain sensors, including silicon nanomembranes [29], silver nanoparticle ink [30], thin films of carbon nanotubes [31,32], graphene films [33,34], 3,4-ethylenedioxythiophene/styrenesulfonate [35], and polydimethylsiloxane (PDMS) based electrically conductive composites such as carbon black [36], graphite [37], carbon nanotubes [38,39], and metallic nanoparticles [40]. In which, PDMS based graphite sensors can detect up to 100% stretch deformation with 50 gauge factors under a strain [36]. In our previous studies, differential air pressure sensors were applied to test the filter rod pressure drop [41] and piezoresistive sensors made of polyester and semi-conductive inks were used in pressure functional assessment of compression stockings [42,43]. Although numerous soft sensors have been explored to monitor skin surface characteristics [44,45], there is no soft sensor that can be applied to structure the morphological pressure map of the dynamic interface pressure that is induced by an IPC device in compression therapy.

The IPC device mimics the muscular function to generate a peristaltic centripetal pressure or sequential gradient pressure waves on the targeted positions of limbs for augmenting venous return. During the interaction between the IPC device and a lower limb, pneumatic pressures in bladders, air mass flow supplied by the IPC device, and the resultant interface pressures existing within the IPC device‒human body system are the key parameters for determining the pressure dosage delivery, compression cycles, and compression therapeutic effects. In this study, to detect the key parameters in the IPC device operation in a timely manner, a novel matrix soft sensor-based monitoring system was designed and developed to detect, record, and display different dynamic interface pressure magnitudes and mappings in real time induced by the IPC device on a continuous skin surface of a lower limb in two-dimensional (2D) and 3D views. The involved matrix soft sensors were fabricated by transferring a nanoforce-sensitive semiconductor ink to a polyethylene terephthalate substrate by using advanced screen-printing technology. The proposed IPC monitoring device demonstrated the applicability to qualitatively and quantitatively illustrate interface pressure magnitudes and distributions with a sustained or an intermittent mode during specific inflation‒deflation cycles. Moreover, the air pressure inside the pneumatic bladder(s) (chamber) was monitored using differential air pressure sensors to establish numerical relationships between dynamic pressure mapping and the pneumatic air pressures of the IPC device. In this new system, Bluetooth was used to wirelessly transmit voltage signals and the converted pressure values to display morphological pressure mapping in a timely manner on the computer screen by using the engineered algorithm-driven software unit.

## 2. Materials and Methods

### 2.1. Materials and Measuring System Setup

**Figure 2:**
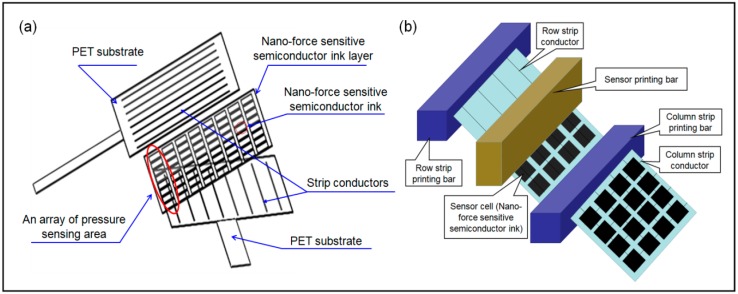
The matrix soft sensor. (**a**) The structure of the soft sensor; (**b**) The fabrication of the soft sensor.

To detect the interface pressure between the IPC and a lower limb, a matrix soft sensor was engineered in a sandwich structure that comprised two polyethylene terephthalate (PET) substrates (size: 125 × 125 × 0.5 mm^3^) and a nanoforce-sensitive semiconductor ink layer (Rouxi Technology company, China) as shown in Figure 2a. To sense the mechanical forces induced by inflatable pneumatic bladders, a nanoforce-sensitive semiconductor ink was transferred to the substrates through an advanced screen-printing technology [46,47]. Sixteen strip conductors were halved to be vertically and horizontally printed on the inner surfaces of the two PET substrates (films), respectively. When the two films were integrated, an array of a pressure sensing area was formed, as displayed in Figure 2b. The size of one sensitive area (i.e., sensor cell area) was 12 (length) × 12 (width) × 1 (thickness) mm^3^. The external pressure applied on the cell areas are reflected by the resistance variation in the semiconductor in the sensing area; thus, the real-time measurement of the intermitted or sustained skin pressure was obtained using the IPC device.

The working mechanisms of the studied matrix soft sensor are analyzed below. When the external pressure is applied to the surface of the soft sensor, the resistance of the soft sensor decreases due to the decrease of both resistance of the semi-conductive ink, and the contact resistance between the strip conductors and the semi-conductive inks. To obtain an accurate pressure reading, the contact resistance is considered here [48]. Then, the total resistance of the soft pressure sensor can be assessed using the below formula:

(1) $$R_{sensor}=2R_{con}+R_{ink}$$

where *R_sensor_* is the total resistance of the sensor; *R_con_* is the contact resistance between the strip conductors and the semi-conductive inks; and *R_ink_* is the resistance of the semi-conductive ink.

By using the Holm formula [49], the contact resistance can be further analyzed in terms of the below equation:

(2) $$R_{con}=\frac{\rho }{2}\sqrt{\frac{\pi H}{F}}$$

where *ρ* is the electrical resistivity of the semi-conductive ink; *F* is the applied force; *H* is the Meyer hardness of the PET substrate of the matrix soft sensor.

Considering that the semiconductor ink is a type of composite material (i.e., conductive filler particles and intrinsic particles), *R_ink_* can be calculated using Equation (3) [50] as follows:

(3) $$R_{ink}=R_{0}(1-\varepsilon )e^{-rD\varepsilon [\pi /{\left(6\phi \right)}^{1/3}-1]}\;r=\frac{4\pi }{h}\sqrt{2m\varphi }\;s_{0}=D[\pi /{\left(6\phi \right)}^{1/3}-1]$$

where *R_0_* is the initial resistance of the semi-conductive ink; *ε* is the strain; *D* is the filler particle diameter; *φ* is the volume fraction of the filler particles; *h* is Plank’s constant; *m* is electron mass; *ϕ* is the height of the potential barrier between two adjacent filler particles; and *S_0_* is the initial distance between the two adjacent filler particles.

In application, the strain could change slightly with time when a constant external pressure is applied to the soft sensor. A single Maxwell or a single Kelvin-Voigt element cannot accurately represent the transient response of the soft sensor. Therefore, a constitutive equation of the standard linear solid model was considered, where the compressive stress component is only of interest, while other stress and strain components would not be taken into account [48,51]. Then, the strain as a function of time can be calculated as follows:

(4) $$\varepsilon (t)=\frac{P}{E_{0}}+\frac{P}{E_{1}}(1-e^{-(E_{1}/\mu_{1})t})$$

where *ε(t)* is the induced strain; *P* is the applied pressure; *E_0_* and *E_1_* are the elastic moduli of the springs; *μ*_1_ is the viscosity of the damper element; *t* is the time.

Therefore, the relationship between the resistance of a semi-conductive ink and the applied external pressure, in respect of the effects of contact and creep, can be expressed as follows:

(5) $$R_{sensor}=\rho \sqrt{\frac{\pi H}{F}}+R_{0}[1-(\frac{P}{E_{0}}+\frac{P}{E_{1}}(1-e^{-(E_{1}/\mu_{1})t}))]\times e^{-rs_{0}(\frac{P}{E_{0}}+\frac{P}{E_{1}}(1-e^{-(E_{1}/\mu_{1})t}))}$$

**Figure 3:**
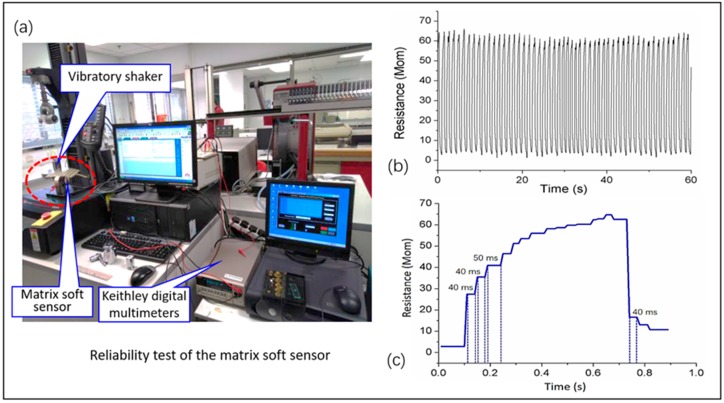
The response analysis of the matrix soft sensor. (**a**) A vibratory shaker and a Keithley digital multimeters system for reliability test of the matrix soft sensor; (**b**) A cyclic weight loading test; (**c**) Analysis of response time.

In this study, the resistance of the matrix soft sensor (R_sensor_) was verified by the experimental measurement. A vibratory shaker and a Keithley digital multimeters system (Figure 3a) were used to characterize the reliability of the soft sensor. Referring to the IPC working time and existing literature on soft sensors [29,30,31,32,33,34,35,36,37,38,39,40,41,42,43], the R_sensor_ was measured under the pressure exerted by a 0.5 kg weight when the vibratory shaker was set at a constant frequency of 1 Hz. The produced electrical signals were collected by the digital multimeters at a sampling period of 10 ms. Figure 3b presents the resistance variation of the soft sensor within 60 s. The response time of the resistance depends on both loading/unloading frequencies of the weight and the sampling periods of the digital multimeters in the test. At a cyclic weight loading, the response time (hysteresis time) of the soft sensor reached 50 ms as shown in Figure 3c. According to the related studies, the response times of PDMS/graphene [52] and ultrathin epidermal piezoelectric sensors [53] are 40 ms and 60 ms, respectively. Compared with them, the current soft sensor shows an acceptable response time towards the applied external pressure. For the studied IPC system, its period of one compression cycle of inflation-deflation is commonly greater than 2 s. The matrix soft sensor, is therefore appropriate to be used to detect interface pressure between the pneumatic bladder and the skin.

**Figure 4:**
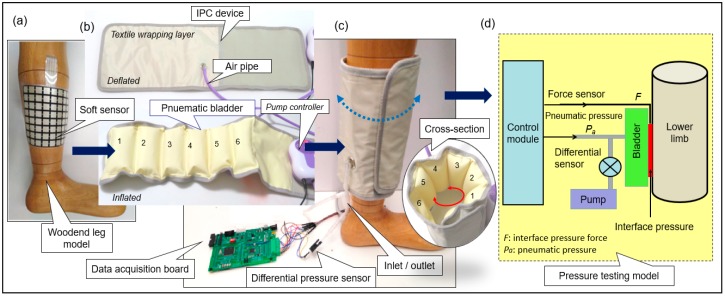

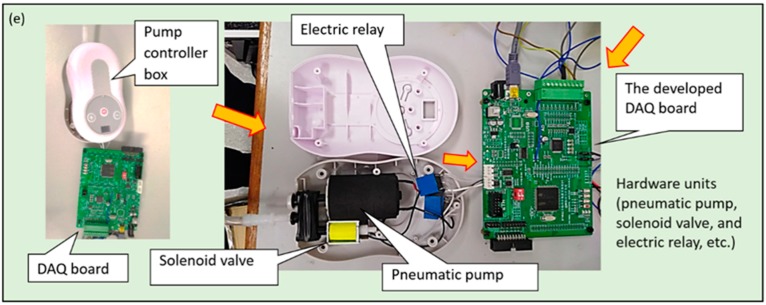
The dynamic pressure monitoring system. (**a**) Matrix soft sensor wrapped on the wooden leg model; (**b**) IPC device in deflation and inflation conditions; (**c**) Monitoring system acting on the wooden leg model with IPC device; (**d**) The developed dynamic interface pressure and pneumatic pressure testing model; (**e**) The developed data acquisition (DAQ) board and the controller box (involving pneumatic pump, solenoid valve and electric relay).

To monitor the dynamic pressures induced by IPC in the inflation‒deflation cycles, the developed soft sensor was mounted onto a wooden leg model, as illustrated in Figure 4. An IPC unit in a mode of MK-940, as a pneumatic compression therapeutic device, was applied to the soft sensor in a wrapping manner. Six bladders each with a size of 19 (length) × 6.5 (width) cm^2^ were evenly set along the wrapping layer. The layer was laminated with a woven-based textile shell with a size of 23 (width) × 70 (length) cm^2^. This laminated wrapping layer formed the major body of the IPC unit and was unfolded onto a wooden leg model to simulate the treatment condition. Moreover, this IPC unit comprised a pneumatic pump, a data acquisition (DAQ) card, matrix soft sensor, a differential pressure sensor, and air pipes. These components were used to form a pump control system for both air source supply and dynamic pressure delivery. Pneumatic bladders and pneumatic pump (model no. KPV04, maximum pressure: 70 kPa, maximum air mass flow rate: 2.0 LPM) were connected using flexible air pipes. One differential pressure sensor (model no. MPX 5100DP) was connected with the air pipes by using tee joints, as shown in Figure 4a. The pneumatic pressure signals collected from the differential pressure sensor were transmitted to the data acquisition card through an analog-to-digital (A/D) converter (a 32-bit microcontroller integrated circuits fabricated by STMicroelectronics‒STM 32) and a data processing unit [54]. Time domain signals of both skin (contact) pressure and pneumatic pressure were real-time displayed on the screen of a laptop. For the designed system presented in Figure 4a–c, a schematic of the pressure testing model was established (Figure 4d). The pneumatic subsystem was an action module without a time control unit. The actions of all the involved hardware components were governed by the developed software. Figure 4e shows the hardware sections involved in the dynamic pressure monitoring system, e.g., the developed DAQ, and the pneumatic pump, solenoid valve and electric relay installed in the controller box.

### 2.2. Data Acquisition (DAQ) System

**Figure 5:**
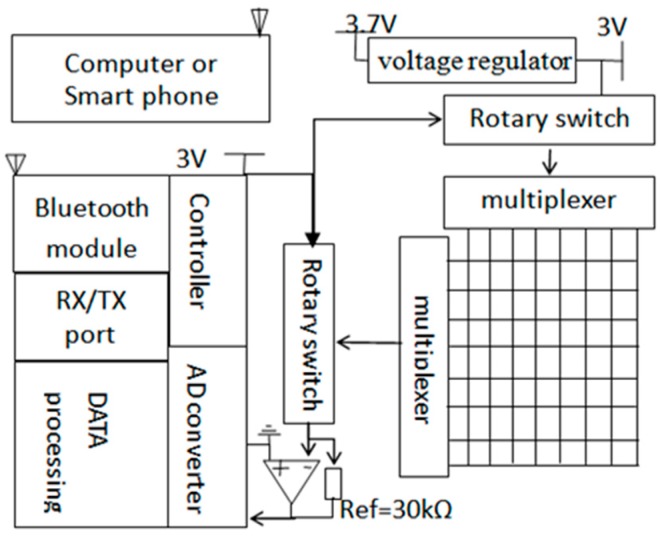
Data acquisition system.

The DAQ system is the core of the IPC dynamic pressure monitoring system and is used to collect pressure signals that are produced by the IPC unit. The unit comprised the Bluetooth module, RX‒TX port, data processing unit, controller, A/D converter, and rotary switches, as shown in Figure 5. A voltage regulator was used to convert 3.7 V of a Li-ion battery to 3 V to be applied as the system voltage. Each cell in the soft sensor was selected by two rotary switches for data collection. The voltage signals of the cells were extracted from the voltage dividers and were sent to the A/D converter channels in STM32. The voltages were then transformed into digital signal values by utilizing a 12-bit A/D converter [54,55]. By using the serial port (RX/TX), the force values were sent to the Bluetooth module, and the Bluetooth antenna set in the Bluetooth module then wirelessly transmitted the force values to a remote receiver (e.g., a smartphone or laptop).

To calculate the pressure voltage data, the sensor array was connected to the reference resister (*R_ref_* = 30 kÙ, based on the sensor resistance) and ground (GND) through a rotary switch and connecting cables. The input analog voltage (*A_input_*) can be calculated by the voltage division equation, i.e.,

(6) $$A_{input}=R_{ref}*V_{cc}/(R_{ref}+R_{sensor})$$

where *V_cc_* is the system voltage; *R_sensor_* represents the cell sensor resistance.

The digital output of the voltage value (*D_output_*) can be calculated by

(7) $$D_{output}=2^{m}*A_{input}/V_{ref}$$

where *V_ref_* is the input full-scale voltage; and *m* is the bit length of *D_output_*. The *V_ref_* of the A/D converter channels can be selected to the microcontroller’s positive supply voltage *V_cc_* by using a software controller.

When *V_ref_* = *V_cc_*, Equation (7) can be rewritten as

(8) $$D_{output}=2^{m}*A_{input}/V_{ref}=2^{m}*V_{cc}*R_{ref}(R_{ref}+R_{sensor})/V_{cc}=2^{m}*R_{ref}(R_{ref}+R_{sensor})$$

**Figure 6:**
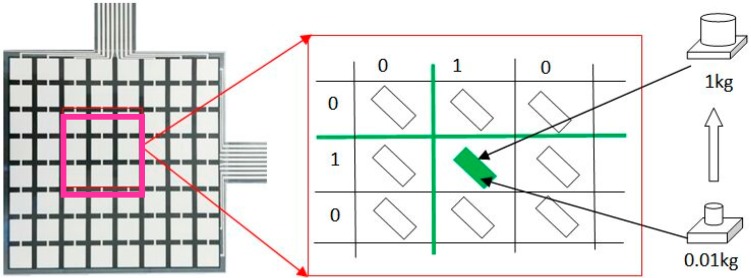
The calibration diagrams of each cell of the matrix soft sensor.

Equation (8) presents the relationship between the digital resistances of A/D converters and the sensor resistances. *V_cc_* did not affect digital outputs. Each cell of the soft sensor was calibrated using different counterweights. The size of the bottom pad of the counterweight was 12 × 12 mm^2^, which fully matched each cell zone based on the standard testing procedures [56,57]. The calibration diagram is illustrated in Figure 6.

**Figure 7:**
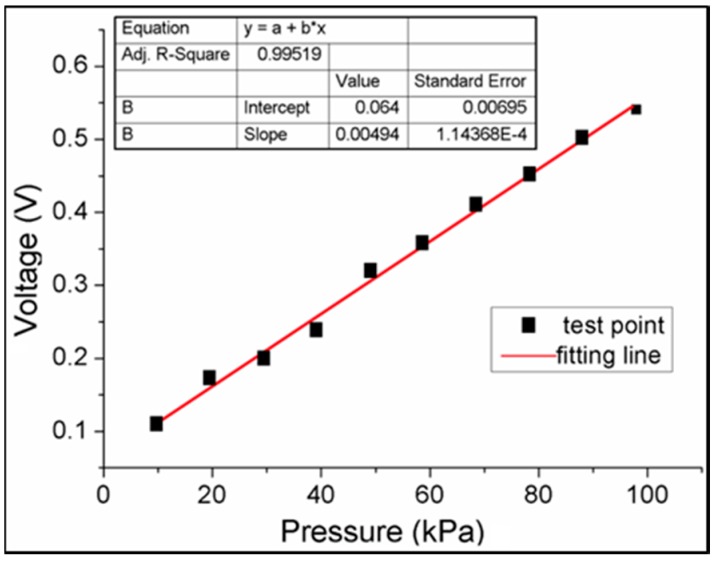
The relationship between the pressure (kPa) and the voltage (V) of the tested sensor cell.

In the calibration, the counterweights were put on each sensor cell one by one in a designed sequence, and the corresponding voltage signals were collected using the developed data acquisition system as aforementioned. Through calibration, a series of counterweights and their corresponding signals were recorded. The relationship between the produced voltages (V) and the pressure (kPa) for each sensor cell shows an approximately linear trend via a curve fitting algorithm [58] (Figure 7). The voltage of the sensor cell increased proportionally with an increase in the external forces, which converted the mass to gravity, that is, g = 9.8 N/kg. This data acquisition system can detect the applied forces from 0.98 N to 9.8 N for each sensor cell (size: 12 (length) × 12 (width) × 1 (thickness) mm^3^). Through the calibration, different sensor cells achieved the same pressure level prior to formal testing.

### 2.3. Software System Setup

**Figure 8:**
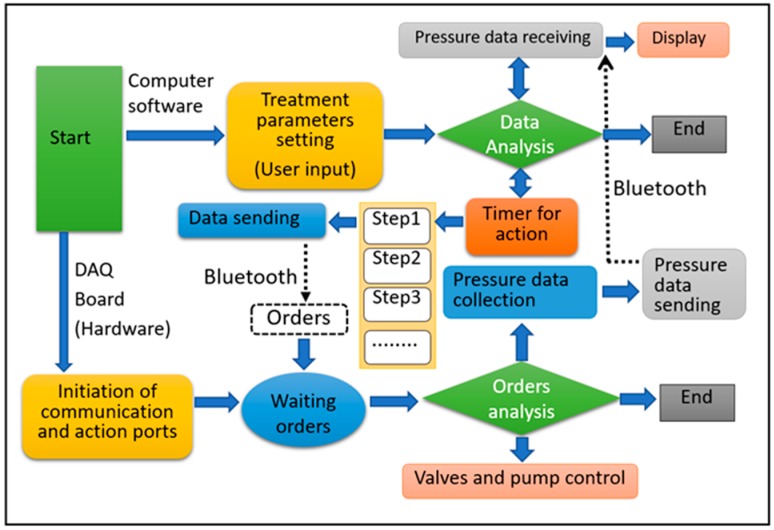
The hard and smooth real-time sections of the developed IPC-lower limb pressure monitoring system.

To visualize the morphological pressure mapping conducted between the IPC unit and the lower limb, a software was designed and developed based on a multi-thread theory for the real-time monitoring [59,60], that allowed users to dynamically observe the biomechanical forces exerted by the IPC unit on the lower limb skin and tissue. Figure 8 illustrates the software and hardware sections of the developed monitoring system, including processes, deadlines, and main scheduling. For the smooth real-time system, the computer software received the data input by the user via the software interface for analysis, where the two processes were initiated. One was data receiving and displaying, and the other was the timer setting (i.e., inflation‒holding‒deflation cycles or treatment period) to control the working of the device. When the DAQ board was launched, the orders sent from the computer software was received by a communication port in an interrupt mode [59,61]. And then, the orders were analyzed to control the action of the pneumatic pump (open or close). Meanwhile, another communication port was open to collect and transmit the data (i.e., pressure) to the software using a scanning method. For the hard real-time system, each order was controlled by the developed software except for the DAQ unit. An embedded technology [54,55] was applied to govern the data receiving or the action of the pneumatic pump in real time. The main deadlines involved in the hard and smooth real-time sections synergistically work to achieve the expected function of the pressure monitoring system.

**Figure 9:**
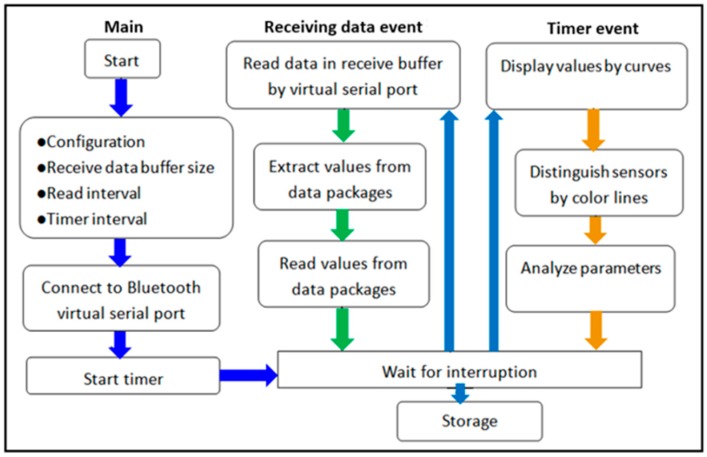
A flowchart of the developed data processing system in the pressure monitoring and display software.

Moreover, the software was used to observe the dynamic variations during pressure dosage delivery and interactions with tissue deformation, thereby obtaining intuitive biofeedback on pressure treatment in practical use. The data processing design of the computer software is presented in Figure 9.

The computers were connected to the remote receiver (i.e., IPC) through Bluetooth. First, an overall configuration including the receiver data buffer size, read interval timeout, and timer interval was set carefully and concertedly to ensure a smooth flow of data reception, display, and processing. Second, after connecting to the Bluetooth virtual serial port, the timer was forced to initiate with interruptions of the waiting status. Two event-driven responses comprising the receiving data event and timer event corresponded to the receiving data and processing data, respectively. For the receiving data event, the sensor values were extracted from the data packages after reading the virtual serial port data buffer and then were transformed to pressure values based on a communication agreement. For the timer event, the sensor values were displayed as time-varying curves in real time. Finally, the program returned to the main body after storing the data.

### 2.4. Working Modes of the IPC Device

The IPC unit was regarded as a primary part to deliver a controllable pressure dosage to the lower limb. The working modes (i.e., inflation‒holding‒deflation cycles) and control logic were governed by the IPC unit. Here, the pressure dosage mode of 23 s‒7 s‒7 s in one compression cycle, which sustains the 37 s treatment condition, was adopted as an example to illustrate a general working mode of the IPC. This working mode includes three stages. First, the inflation process is included in the first 23 s. In this process, the pneumatic pump controller was operated, and the bladder was charged with a constant pneumatic air mass flow. This procedure allowed the pneumatic pressure inside the bladders to gradually increase. Second, the holding process was conducted from 24 to 30 s. In this process, the pneumatic pump stopped working, and the valves closed to maintain the pneumatic pressure inside the bladders for 7 s. Finally, the deflation process was conducted in the final 7 s. Here, the valves were opened to deflate the bladder pneumatic air.

By setting working modes in the testing system, the periodic working rhythms of the IPC unit can be detected by determining the varying amplitudes of the pneumatic pressures. Here, the amplitudes were governed by the pneumatic pump controller and the air valves. The pneumatic pressure variations can be used to determine the pneumatic air mass flow rate under different inflation‒deflation cycles, and to analyze the interactive mechanism of the design parameters of the IPC unit, and to validate the developed IPC monitoring system. Under IPC operation, the pneumatic air was pumped into the bladder and the resultant interface pressure was produced and exerted on the limb skin and tissue. The interface pressure values increased with an increase in the air mass flow rate, expansion of the pneumatic bladder, and acting forces supplied by the IPC. When the deflated valves open, the pneumatic pressure of the bladders and the mechanical force exerted on the skin disappeared. In this system, the control and monitoring ports were the two separated parts. The time-domain dynamic pneumatic pressure and the skin force pressure were monitored in real time and displayed on the screen of the terminal unit. The software interface revealed and specified the quantitative and qualitative relationships between the pneumatic pressure inside the bladders and the interface contact pressure on the skin surface.

## 3. Results and Discussion

### 3.1. Pressure Monitoring Interface

**Figure 10:**
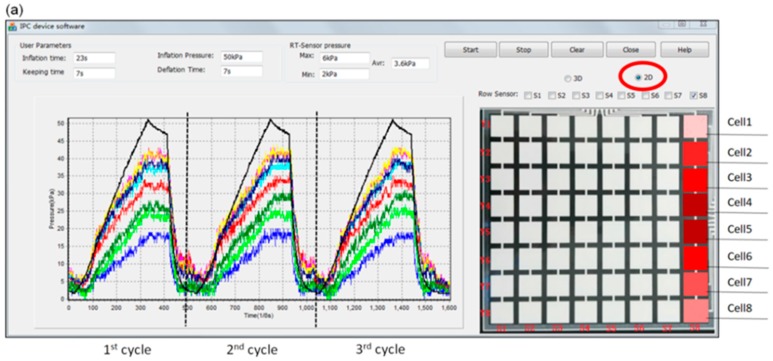

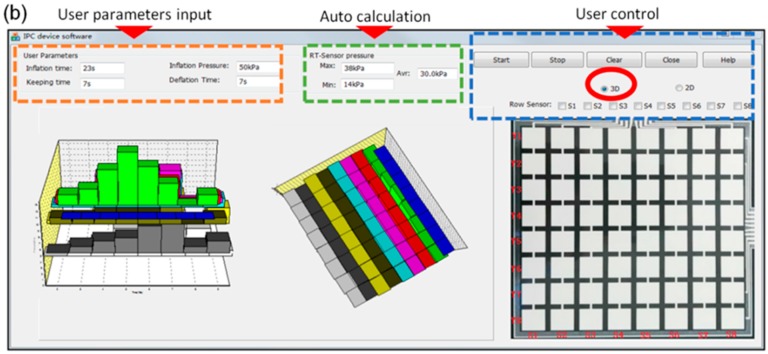
The user interface displaying the developed software of IPC dynamic pressure monitoring system. (**a**) 2D display on the detected skin (interface) pressure along one column of the soft sensor array in three inflation-deflation cycles; (**b**) 3D real-time display of all cells (64) (eight columns of the soft sensor array) and partial cells in colorful tetrahedrons.

In this study, a multi-functional software interface was developed to monitor, visualize, and record data details collected via Bluetooth in real time (i.e., skin contact pressures and pneumatic pressure inside bladders). The user-defined input parameters (i.e., treatment pressure dosages, inflation time, holding time and deflation time) and real-time output parameters (i.e., maximum (peak) pressure, average pressure, minimum pressure, and pressure morphological mapping detected by the engineered soft sensor) were set in the interface of the developed morphological pressure mapping system. Moreover, the variations in the skin pressures sensed by each row and line cells of the soft sensor array were simultaneously displayed in the interface of the software with changes in the pneumatic air supplied by the pump controller in the inflation‒deflation cycles. As shown in Figure 10a, the skin pressure signals sensed by the eighth column (i.e., the highlighted red cells) in the sensor array, which was induced by the bladders of IPC device, were displayed using the color curves during multi-inflation‒deflation compression cycles. Figure 10b illustrates the real-time skin pressure signals sensed by all the cells and the selected partial cells in the matrix soft sensor array induced by the bladders of the IPC unit in color tetrahedrons.

An intermitted pressure mode of 23 s‒7 s‒7 s input by the user on the software interface (Figure 10) was adopted as an example to verify the workability of the designed monitoring system. Here, the developed software and hardware sections (Figure 8) integratively work to fulfil this required compression therapeutic recipe.

Firstly, the developed software received the input parameters by the user for analysis and the DAQ board initiated the ports for receiving the orders. Then, the software opened the port for receiving data and setting the timer for controlling the compression cycle and treatment time (i.e., step 1: 23 s, step 2: 7 s, and step 3: 7 s in one cycle). When the DAQ board received the first order (i.e., step 1: 23 s) from the software and the chip timer collected the pressure data from the soft sensor, a real-time pressure monitoring process started. The pneumatic pump worked for 23 s to inflate the bladder with a constant pneumatic air mass flow. At the same time, the software created the lines or morphologic mappings to display pressure profiles in the software interface based on the received data.

Secondly, following the timer control order (i.e., step 2: 7 s), the pneumatic pump stopped working, and the valves closed to maintain the pneumatic pressure inside the bladders for 7 s. Then, the deflation valves opened in the next 7 s (i.e., step 3) to allow the pneumatic pressure inside the bladders and the contact pressure on the skin surface to release till disappear accordingly.

Finally, one compression cycle was completed and followed by the next cycles repeatedly. In this system, the control and monitoring ports were two separated parts to ensure the real-time monitoring smoothly. The quantitative and qualitative pneumatic compression, skin pressure and their dynamic relations were displayed on the screen of the terminal unit as shown in Figure 10. These relationships largely help designers and engineers of compression devices to efficiently control the pneumatic pump and pressure delivery rhythms and also enable users or physicians to appropriately adjust pressure dosage duration for achieving a high-performance customized compression therapy and user compliance.

### 3.2. System Mechanical Analysis

**Figure 11:**
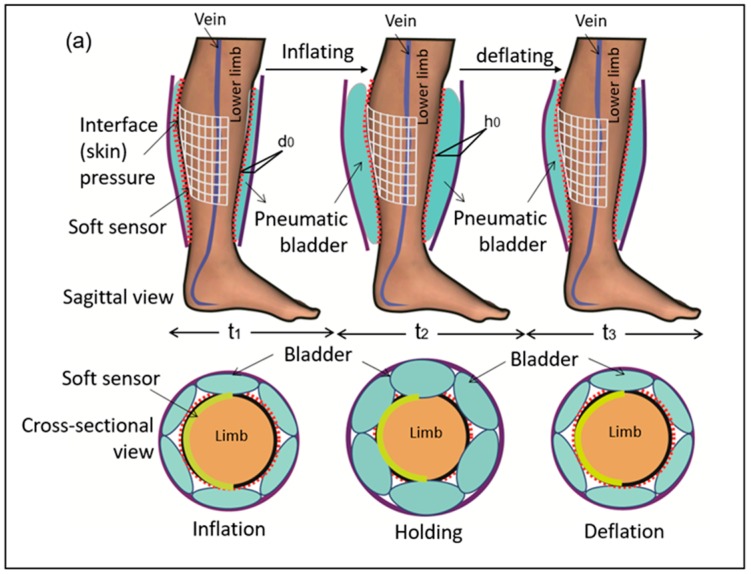

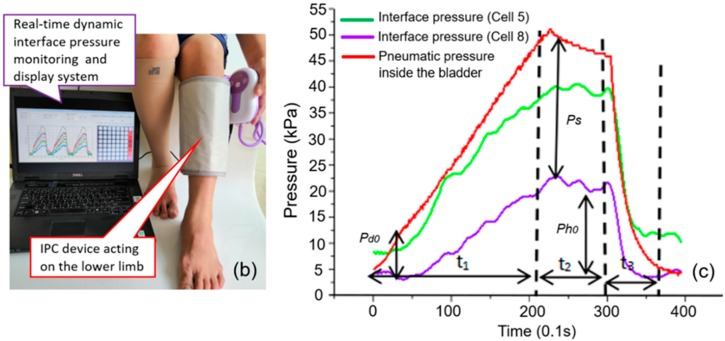
(**a**) The sagittal and transverse views of the bladder and the lower limb system in deflation-holding and deflation cycle; (**b**) Dynamic pressure monitoring system under IPC-lower limb action in practice. (**c**) Variation curves of the integrated interface pressures and pneumatic pressures detected by the developed IPC dynamic pressure monitoring system in one compression cycle.

**Figure 12:**
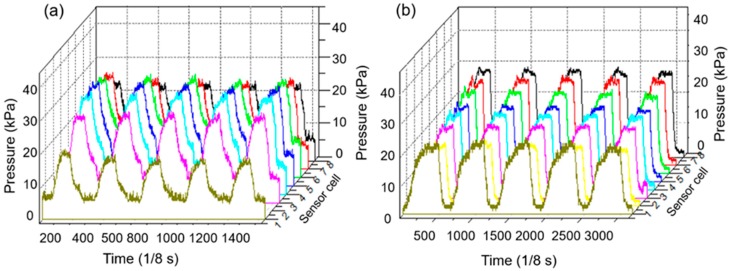
The interface pressures detected by one column of the sensor array under 5 continuous fast and slow compression cycles in treatment. (**a**) Faster compression cycles; (**b**) Slower compression cycles.

Figure 11a illustrates a cross-sectional view of the pneumatic bladder and the lower limb system in the sagittal and transverse planes during the initial inflation-holding-deflation process. Here, *d_0_* indicates the initial thickness of the bladder at the initial inflation stage and *h_0_* represents the thickness of the expanded bladder at the maximum inflation or holding stage. Figure 11b depicts the practical wearing status of the IPC-lower limb system in use. To reflect the dynamic variation in both interface pressure signals and corresponding pneumatic pressure inside the bladder, the integrative results of the real-time skin (interface) pressures detected by the sensor cells S (5) and S (8) that are located in the eighth column of the sensor array and the corresponding pneumatic pressures inside the bladders supplied by the pump system of the IPC device were obtained (Figure 12c). Here, *t*_1_, *t*_2_, and *t*_3_ indicate the inflation, holding, and deflation times of the bladder, respectively. *P_s_* presents the pressure gap between the detected skin pressures and the pneumatic pressure inside the bladders. *P_d0_* presents the pressure gap between the contact skin pressures and the pneumatic pressures inside the bladders at the initial inflation stage, and *P_h0_* presents the pressure gap between the skin pressures and the pneumatic pressures inside the bladders at the holding stage of the compression treatment.

It can be seen that the skin pressures altered due to variation in the corresponding pneumatic pressures in a similar trend profile within one compression cycle. However, the values of skin pressures were lower than those of the pneumatic pressures in general. The energy produced by the inflated airtight bladder not only has to counteract the resistance of the bladder shell for expanding, but also has to deform the soft tissue to transform the aerodynamic force to the biomechanical force, thus promoting hemodynamics for venous return. Contact condition between the IPC bladders and the lower limbs largely influence the displacement (deformation) of contact interface because the human lower limbs are irregular in the cross-sections with varying surface curvatures and tissue stiffness, for example, the tibia bone at the anterior calf with a less radius of curvature (ROC) but higher stiffness, and the gastrocnemius located at the posterior calf with a greater ROC but lower stiffness. Such anatomic geometrics and biomaterials vary the interface pressure synergistically in the compression cycle. In addition, the pneumatic energy inside the bladder may be lost during the transmission process due to potential leakage, material fatigue, or consumption in the expansion on the opposite wall far from the skin surface. These could also be the potential reasons for the resultant lower skin pressures.

### 3.3. Morphological Pressure Mapping

Figure 12a,b illustrates the results of the interface pressure‒time recording by using one column cells of the developed soft sensor under the action of a pneumatic bladder of the IPC device in the five continuous compression cycles and in different working modes of 23 s‒7 s‒7 s and 40 s‒20 s‒20 s, respectively. The developed soft sensor and pressure monitoring system can detect the different inflation dynamics in real time when the maximum interface pressure is set at 150 mmHg (i.e., 20 kPa) as an example. Numerous studies have investigated the effects of different pressure magnitudes and inflation times on venous thromboembolism prophylaxis and ulcer treatment [62,63,64,65]. A higher pressure (120–160 mmHg) and rapid inflation (1–2 s) pneumatic compression of the IPC device produced significantly higher venous velocities in the popliteal and femoral veins compared with the lower pressure (40–50 mmHg) and slow inflation (11–12 s) pneumatic compression, which may offer additional protection from thrombotic complications due to an improved hemodynamic response in both healthy volunteers and patients who were post-thrombotic [62,63]. However, higher pressures (e.g., 120 mmHg) and longer time (e.g., 50 s) might generate effective tissue fluid pressures and sufficient time for moving the stagnant fluid [65]. Intuitively displaying and recording interface pressure‒time variations under the interaction of the IPC device with biologic tissues are highly helpful in analyzing the fluid mechanical effects of IPC on lower limbs in a customized treatment in practice.

**Figure 13:**
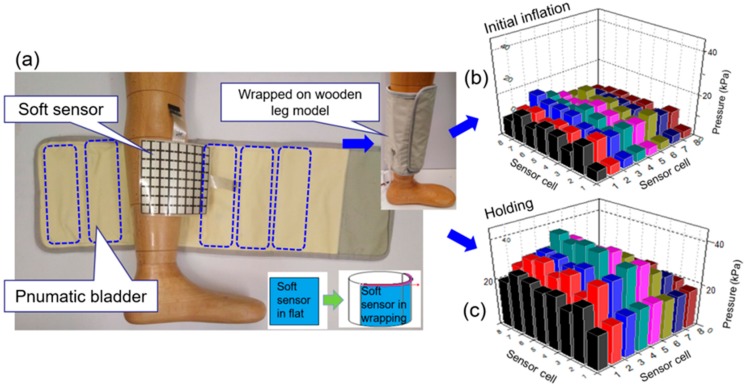
(**a**) A wooden leg model wrapped by the developed matrix soft sensor, (**b**) Morphological pressure mapping at initial inflation and at (**c**) holding stages of one compression cycle by using the IPC device.

Figure 13a‒c illustrates the dynamic morphological pressure mapping when the soft sensor array is unfolded and wrapped around the 3D rigid leg model with a round cross-section. The curved surface of the leg model varied the pressure matrix profile, thus presenting uneven pressure pillar distributions at the initial inflation and holding stages of the IPC device operation. Interface pressures gradually reduced from the contact center of the sensor array (e.g., cells 4 and 5) to the two ends (e.g., cells 1 and 8) due to the closeness variation between the bladders and the lower limb in the compression cycles. Based on the visual and dynamic pressure matrixes, the pressure effects of bladder design can be analyzed and adjusted in terms of the different acting zones to achieve the expected dynamic pressure function.

**Figure 14:**
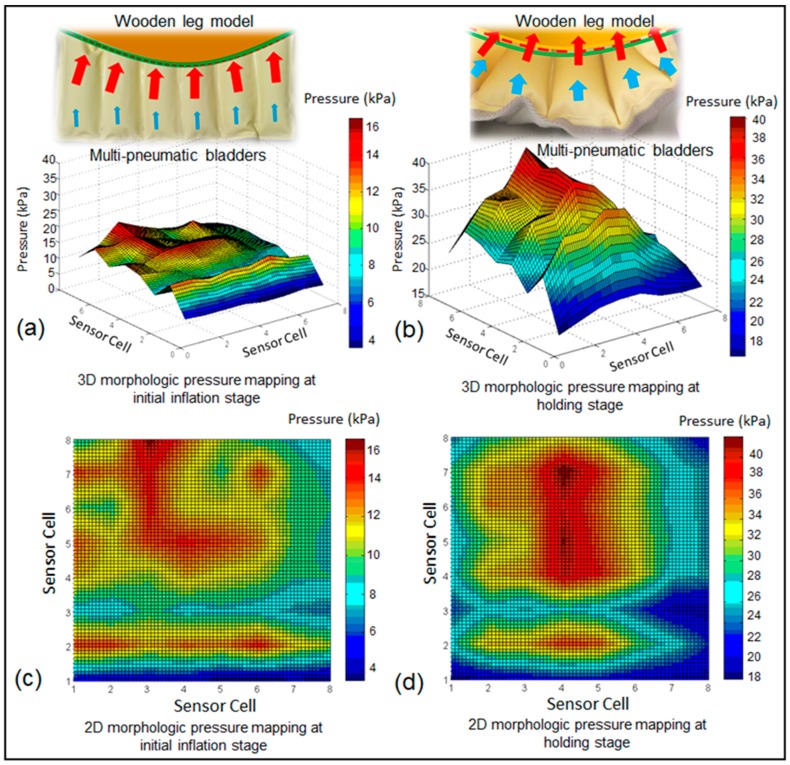
The 3D and 2D morphological pressure mapping detected by the developed dynamic interface pressure monitoring system at different stages of compression cycles by the IPC in vitro. (**a**) 3D morphologic pressure mapping at initial inflation stage, (**b**) 3D morphologic pressure mapping at holding stage, (**c**) 2D morphologic pressure mapping at initial inflation stage, (**d**) 2D morphologic pressure mapping at holding stage.

**Figure 15:**
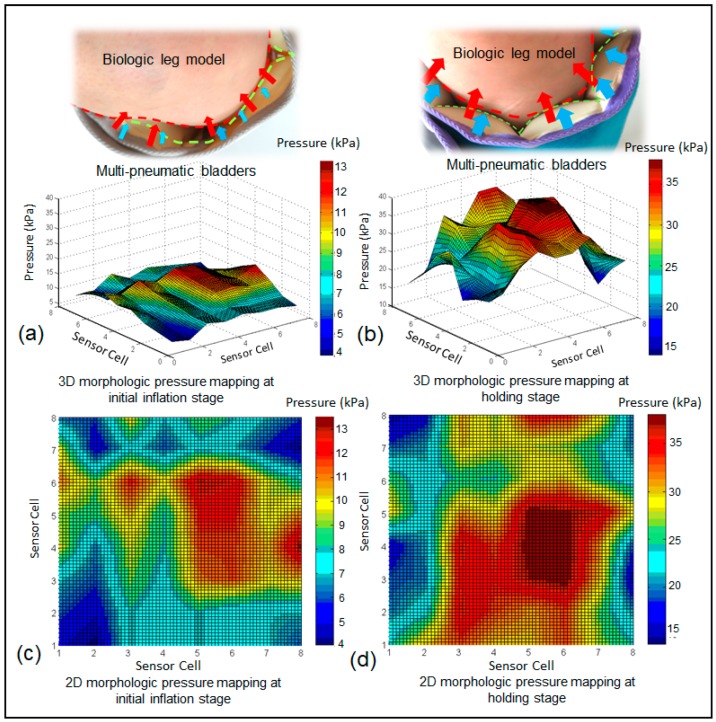
The 3D and 2D morphological pressure mapping detected by the developed dynamic interface pressure monitoring system at different stages of compression cycles by IPC in vivo. (**a**) 3D morphologic pressure mapping at initial inflation stage, (**b**) 3D morphologic pressure mapping at holding stage, (**c**) 2D morphologic pressure mapping at initial inflation stage, (**d**) 2D morphologic pressure mapping at holding stage.

Figure 14a‒d further describes the morphological pressure mapping in 3D and 2D nephograms when the IPC device adopts the inflation mode of 23 s‒7 s‒7 s in vitro. Continuous color mapping reveals a more vivid morphological pressure fluctuation by the IPC. The peak skin pressures (wave crest) were observed in the areas at which the inflated bladders were in contact with the skin during the holding period. The distributions of the pressure wave crest and trough are relatively even when the IPC device was mounted on the rigid wooden leg model compared with the pressure waves when the IPC unit was positioned on the biologic lower limb in vivo (Figure 15). 

It can be seen that (Figure 15), the deformed soft tissue induced by the expansion of the bladders led to an increased contact area of the peak pressure zones and weakened periodic pressure wave crests. The highest interface pressures were found at the gastrocnemius (posterior leg) and gradually decreased to the lateral and medial sides under the IPC device in the tested wrapping condition. The human lower limb is irregular in cross-sections and heterogeneous in tissue stiffness. This characteristic alters the rhythmic interface pressures obtained using IPC, as shown in the in vitro test. Further studies will be conducted to investigate morphological pressure mapping of the pneumatic compression device when applied to lower limbs with different dimensions and biomaterial properties (e.g., younger users, the elderly, and athletes) in different wearing environments.

## 4. Conclusions

In this study, a dynamic interface pressure monitoring system was designed and developed to detect and visualize interface (skin) pressures induced by an IPC device in 2D and 3D morphological mapping. A sandwich-structure matrix soft sensor was used as the core element with the developed hardware and software systems to demonstrate the real-time monitoring ability and to vividly display the sustained and intermittent pneumatic pressure by using IPC for different inflation‒deflation compression cycles. The dynamic interface pressures in 2D and 3D views, the quantitative pneumatic pressures inside the bladder, and their dynamic relations were integrated to simultaneously display in the designed interface of the proposed dynamic pressure monitoring system. The system can not only detect specific pressures at the targeted zones but also conduct morphological pressure mapping in a continuum interface. The novel dynamic pressure monitoring system presents the favorable capability of intuitively reflecting biomechanical forces and their variations under rhythm pressures by IPC. This capability provides valuable technical guidance for performing parameter design of the high-performance pneumatic compression device. The IPC device design can be realized by visualizing, quantifying, and real-time analyzing the variations and relations of the contact condition of the IPC and lower limb, pneumatic pump control, and bladder morphologies, thus improving compression therapeutic efficacy and fulfilling personalized demands in daily treatment.
